# Improving diaper design to address incontinence associated dermatitis

**DOI:** 10.1186/1471-2318-10-86

**Published:** 2010-11-22

**Authors:** Anne-Marie Beguin, Evelyne Malaquin-Pavan, Claudine Guihaire, Anne-Marie Hallet-Lezy, Sandrine Souchon, Vanessa Homann, Petra Zöllner, Maximilian Swerev, Rüdiger Kesselmeier, Fridmann Hornung, Hans Smola

**Affiliations:** 1 Hôpital C. Celton (APHP), 4 Parvis C. Celton 9, F-2130 Issy les Moulineaux, France; 2 Hôpital Vaugirard (APHP), 10 rue Vaugelas, F-75015 Paris, France; 3 Paul Hartmann AG, PO Box 1420, D-89504 Heidenheim, Germany; 4 Department of Dermatology, University of Cologne, D-50924 Cologne, Germany

## Abstract

**Background:**

Incontinence associated dermatitis (IAD) is an inflammatory skin disease mainly triggered by prolonged skin contact with urine, feces but also liberal detergent use when cleansing the skin. To minimize the epidermal barrier challenge we optimized the design of adult incontinence briefs. In the fluid absorption area we interposed a special type of acidic, curled-type of cellulose between the top sheet in contact with the skin and the absorption core beneath containing the polyacrylate superabsorber. The intention was to minimize disturbance of the already weak acid mantle of aged skin. We also employed air-permeable side panels to minimize skin occlusion and swelling of the stratum corneum.

**Methods:**

The surface pH of diapers was measured after repeated wetting with a urine substitute fluid at the level of the top sheet. Occlusive effects and hydration of the stratum corneum were measured after a 4 hour application of different side panel materials by corneometry on human volunteers. Finally, we evaluated skin symptoms in 12 patients with preexisting IAD for 21 days following the institutional switch to the optimized diaper design. Local skin care protocols remained in place unchanged.

**Results:**

The improved design created a surface pH of 4.6 which was stable even after repeated wetting throughout a 5 hour period. The "standard design" briefs had values of 7.1, which is alkaline compared to the acidic surface of normal skin. Side panels made from non-woven material with an air-permeability of more than 1200 l/m^2^/s avoided excessive hydration of the stratum corneum when compared to the commonly employed air-impermeable plastic films. Resolution of pre-existing IAD skin lesions was noted in 8 out of 12 patients after the switch to the optimized brief design.

**Conclusions:**

An improved design of adult-type briefs can create an acidic pH on the surface and breathable side panels avoid over-hydration of the stratum corneum and occlusion. This may support the epidermal barrier function and may help to reduce the occurrence of IAD.

## Background

Worldwide more than 200 million persons suffer from severe forms of urinary and/or faecal incontinence and a significant number ranging form 22% to 26% report mild urinary incontinence problems [1]. Population-based studies reported a prevalence of moderate to severe urinary incontinence of more than 42% in over 60-year old women [2]. While the true prevalence is unknown, some epidemiological studies suggest a frequency of faecal incontinence of 2.7% (daily) 4.5% (weekly), and 7.1% (once per month or less) in individuals 18 yr or older in the outpatient setting [3]. The symptoms of incontinence can be treated with a wide range of conservative therapies, surgery and drugs (reviewed in [4,5]). However, in many patients these therapies fail to some extent and incontinence impairs the quality of life and is extremely embarrassing for the patients affected.

Absorbent incontinence products are the mainstay if all other therapies have failed. Their basic technology is derived from baby diapers [6,7] while design and additional features are specifically adapted to adults. For example fluid absorption capacity reflects the larger urinary volumes in adults, the outer cover materials are selected to avoid rustling noises and the anatomical shapes are designed for maximal fit and wearing comfort [8,9]. While technological development continually improves the performance [10], surprisingly little attention has been focused on the skin-friendliness in adult-type of absorbent incontinence products. This neglects an overt clinical problem commonly referred to as incontinence associated dermatitis (IAD). IAD is an irritant or contact type of skin inflammation of the perineal or perigenital region and needs to be clearly distinguished from cutaneous type IV allergies [11,12]. IAD is reported to affect incontinent patients from 5.7% to more than 42% [13,14]. These large variations may reflect that different populations were analyzed and were at different risk to develop IAD. IAD appears to be strongly associated with age and this very well correlates with a more fragile epidermal barrier and a reduced capacity of the skin to regenerate and repair.

Many factors contribute to the barrier function of skin. For the outermost part of the epidermis, the stratum corneum, the "brick and mortar" model was proposed by Elias [15] which describes the roles of complex extracellular lipid structures (mortar) and protein-rich corneocytes (bricks). Both components are essential for the formation of a robust epidermal barrier. The epidermal barrier is a dynamic structure which is continuously renewed from below and dissolved on the outermost surface through controlled desquamation. The proliferation rate of basal cells, suprabasal keratinization processes, compaction of the corneocytes and stabilization of the intercellular adhesion structures through transglutaminases are taking place in epidermal parts below the epidermal barrier while proteolytic activity in the outermost parts of the stratum corneum regulates the desquamation process (reviewed in [16]). All these events are tightly controlled and the epidermal barrier function is critically dependent on the balance of all processes involved.

With age, the epidermal barrier becomes more susceptible to external stress. The stratum corneum is less acidic [17] causing a reduced epidermal lipid synthesis as well as a delay in lipid-derived lamellar membrane maturation [17]. Moreover, enzymatic systems with specific functions in different parts of the stratum corneum are perturbed weakening the epidermal barrier from a functional aspect [18]. As a result external stress leads to defects in the epidermal barrier and due to a reduced repair capacity in aged skin, inflammation is initiated. Skin contact with faeces, alkaline urine after decomposition on the skin or in urinary tract infections with urease producing bacteria [19] and the liberal use of skin cleansing products [20] provide a plethora of strong, harmful stimuli stressing the epidermal barrier and giving rise to IAD in extreme situations.

Intensive skin care and hygiene, several times per day including cleansing, washing and drying are mandatory to care for these patients. Several skin care regimens have been compared and may help to reduce the prevalence of IAD [21,22]. The variables to consider are clinical effectiveness but treatment-cost-per-day should not be underestimated as the latter has a large influence on the affordability and availability for patients. In terms of clinical effectiveness, a recent study of four different skin care regimens showed that the rate of IAD was lowered in all of the four skin care protocols [21]. Another important variable to prevent IAD are absorbent incontinence products in the appropriate size and conforming shapes [8,9]. This topic is poorly reported in the literature as most of the attention has been focused on technical aspects such as the absorptive capacity of the different incontinence briefs for adults.

Historically, the introduction of polyacrylate superabsorbing polymers in baby diapers resulted in a dryer skin microenvironment. These polymers compartmentalize liquids and prevent re-wetting even under pressure [6,7,23,24]. Clinical studies showed a reduced incidence of diaper rashes [6,7,23,24]. While the surface pH did receive special attention in these studies [6], own unpublished results indicate that the surface pH of several baby diapers is still in the range between 5.56 to 6.27 which possibly reflects the need of a pH range around 6 - 7 best for optimal utilization of the polyacrylate superabsorber and its fluid absorption capacity. These polymers are standard now in almost all absorbing incontinence products and absorption capacities have become similar in most of these products. While the skin acid mantle is known for a long time [25], new findings on the molecular biology of the epidermal barrier formation have renewed the interest in the acidic surface pH of the skin [17,26]. Hence we aimed at constructing a diaper which better accommodates the special requirements of the epidermal barrier. We wanted to achieve a skin-adapted, acidic pH at the surface of the diaper in contact with the skin when wet. Moreover, we wanted to reduce the occlusive effects and the negative impact on the epidermal barrier by altering some of the materials employed.

The aim of this study was to test how the modified design affects parameters of the epidermal barrier. We resorted to a range of non-invasive skin measurements such as surface pH measurements on products and corneometry [27] on the skin of volunteers. In addition, we describe details of this brief design in a series of IAD patient histories where these briefs had been utilized to support clearance of IAD lesions.

## Methods

### Patients

During the institutional switch to new, skin-adapted incontinence briefs, the skin state of 12 patients was documented for 3 weeks. These patients suffered from IAD and were in long-term and rehabilitation care. The patients consented to the recording of their data. This series of case reports is exempt from ethics committee approval as confirmed by the Freiburg IRB (Freiburg Ethikkommission International, Freiburg, Germany). The evaluation of the clinical data strictly adhered to the spirit of the Helsinki Declaration. In the institutions specialist nursing clinicians were nominated as consultants to deal with IAD. In cases where the skin lesions were so severe or difficult to interpret, dermatologists were invited to review the skin symptoms (Beguin et al., manuscript in preparation). The incontinence brief covered skin was evaluated for presence of erythema, infiltrated erythema (dry or exuding), pustules (intact or ruptured) or signs indicative for infection. To measure the severity of skin lesions we used a score reflecting the severity of epidermal barrier disruption and clinical signs of infection (signs of infection = 5 points, pustules = 4 points, infiltrated exuding erythema = 3 points, infiltrated dry erythema = 2 points, erythema = 1 point, normal skin = 0 points). The left and right body sites were recorded separately. For the skin score the most severe lesion type in each patient was considered for evaluation. The size of the lesions was measured with the largest and smallest diameter whenever possible and the lesions were not considered to be disseminated. The skin state was recorded weekly for 3 weeks. This scoring system was designed to facilitate description and reporting of the skin state, the number of patients was insufficient to validate the score per se. Descriptive statistics, t-test, were used to compare changes during the observational period. A *p*-value <0.05 was considered to be statistically significant.

### Development of skin-adapted incontinence brief

**Figure 1:**
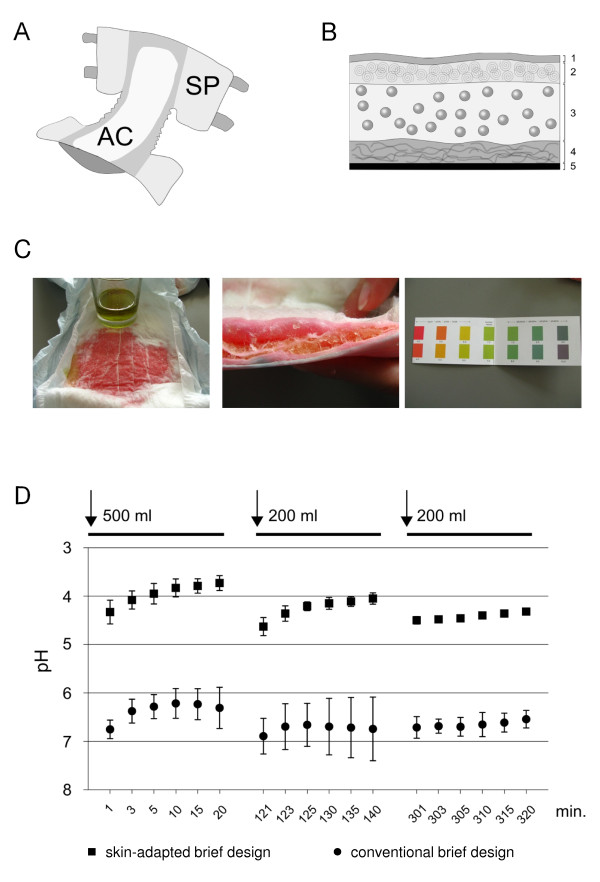
**Development of a skin-adapted absorbent core for adult-type briefs**. **A: **Illustration of a generic diaper design. AC corresponds to the absorbent core, SP corresponds to the side panels. Side panels can be made from plastic films (including micro-perforated films) which are virtually impervious to air or from nonwovens with extremely high air-permeability characteristics. **B: **Schematic illustration of a "skin-adapted" incontinence brief design. 1 - corresponds to the top sheet which is in direct contact with the skin; 2 - curled fiber cellulose layer; 3 - standard cellulose fluff containing superabsorbent polymers; 4 - cellulose distribution layer to facilitate fluid distribution and the utilization of superabsorbent polymers which did not have had direct initial contact with fluid/urine; 5 - outer sheet. **C: **pH gradient within the skin-adapted product design. A colored pH indicator clearly demonstrates the gradient inside the product. Within minutes after wetting the curled fiber layer stains intensively red indicating an acidic environment on the skin facing surface (left picture). Inside the brief the superabsorbent polymer-containing fluff stains yellow to green (middle picture) which indicates a pH where the superabsorbent polymer has its absorption optimum. This internal gradient is stable for several hours. Red corresponds to pH values around 4.0, yellow to a pH around 6.0, green to a pH around 7.0 (right picture). **D: **Surface pH measurements after repeated loading of the incontinence brief. 500 ml 0.9% NaCl solution (urine replacement solution) with a starting pH of 6.8 were rapidly applied (within 30 seconds) to the absorption core of the briefs. pH measurements were taken after 1, 3, 5, 10, 15 and 20 minutes. To simulate multiple micturition, the brief was left untouched and loaded with 200 ml of the fluid after 120 minutes and after 300 min. Time of pH value readings are indicated on the x-axis, the y-axis indicates the pH value. Black squares correspond to the surface pH of the skin-adapted incontinence brief design; black circles correspond to the conventional design of incontinence briefs. The experiments were done in triplicates in two independent series.

To achieve a skin-neutral pH value between 4.5 and 5.5 on the surface of the incontinence brief, the design and the materials of the absorbent core had been changed (Figure 1A). Curled fiber (CMC 525, Weyerhaeuser International Inc., Geneva, Switzerland) a citric acid-crosslinked and a specially processed type of cellulose fiber [28], was interposed between the lining sheet in contact with the skin and innermost superabsorbent polymer (SAP)-containing cellulose fluff. Curled fiber is modified in an citric acidic environment and this favors the formation of cross-links to maintain a twisted and curled fiber architecture. This type of fiber has soft haptics and an excellent ratio between fluid holding and distribution characteristics. Beneath the curled fiber layer the normal cellulose and SAP containing core binds the absorbed fluid and reduces the re-wetting at the level of the skin. SAP can absorb up to 80 - 100 times its own dry weight of water, and 20 - 50 times of salt-containing fluids such as urine. Typically the maximum fluid absorption of SAP is at pH values around 7.0 - 8.0. To utilize the entire amount of SAP inside the absorbent pad, fluid needs to be distributed within the pad over larger distances. This is achieved by a third cellulose-containing layer beneath the outer cover material.

After soaking of the skin-adapted incontinence brief with water or a urine surrogate solution (0.9% NaCl solution, pH 6.8) an internal pH gradient builds-up. To illustrate the pH gradient within the incontinence brief a pH indicator (Unisol 410, Macherey-Nagel, Düren, Germany) containing-solution (indicator stock solution diluted 40 drops/100 ml 0.9% saline solution) was used. After equilibration for 5 minutes the absorbent brief was cut through and the different colors indicated the pH value in the different zones of the product. The exact pH on the surface and inside the core of the briefs was further investigated with a pH Meter CG841 from SCHOTT with a Mettler Toledo In Lab^® ^Surface electrode. The device was calibrated prior to usage. Incontinence briefs (skin adapted design - MoliCare Premium, Paul Hartmann AG, Heidenheim, Germany; conventional product - Tena Comfort Plus, SCA Hygiene Products AB, Göteborg, Sweden, as one of the most sold products with a "classical" design) were loaded within 30 seconds with 500 ml of a 0.9% saline solution (0.9% NaCl solution, pH 6.8) corresponding to approximately 50% of the maximum absorption volume of the incontinence brief. The surface pH was measured 1, 3, 5, 10, 15 and 20 min. after addition of the saline solution. Longer time spans could not be followed as the surface became so dry that reliable measurements became impossible. Incontinence briefs were left at room temperature for 2 hours (from the start of the experiment) before renewed loading with another 200 ml of the saline solution (0.9% NaCl solution, pH 6.8, loaded within 30 seconds) corresponding to an additional 20% of the maximum absorption volume. The surface pH was measured as above. After 5 hours (from the start of the experiment) the incontinence briefs were finally moistened with final 200 ml of the saline solution (0.9% NaCl solution, pH 6.8) so that approximately 90% of the maximum absorption capacity were exhausted. The surface pH was measured after 1, 3, 5, 10, 15 and 20 min after fluid addition. The measurements were performed on 3 briefs each in two independent experiments and the mean and standard deviation are shown for each time point.

### Breathability of non-woven side panels

To avoid occlusion and maceration of the skin, breathable, non-woven side panels were included in the design of skin-adapted incontinence products. Physical air pass-through was measured according to the standardized method outlined in "Standard Test Methods for the Nonwovens and Related Industries, INDA, EDANA WSP 70.1 (2005)" [29] in three samples with two repeats. Water vapor transmission was measured according to the ASTM E398 method [30] in three representative samples with two repeats. Occlusive effects on the skin were determined on 13 healthy human volunteers (9 males and 4 females, age_(mean ± SD) _50.6 ± 2.1 years) consenting to the procedures which were performed under supervision of a dermatologist. The experiments were only performed once as intra-individual i.e. seasonal fluctuations are known for some constituents of the epidermal barrier [31]. 4 × 4 cm samples of non-woven and plastic film material each were applied on the same volar forearm for 4 hours. After removal of the samples, skin conductance was immediately measured with a Multi Probe Adapter MPA 9 device (Courage + Khazaka, Cologne, Germany) fitted with a corneometer probe (Corneometer CM 825) on non-treated adjacent skin (control), and on non-woven or plastic film covered skin. Values are shown as a difference of corneometry readings at the end of the observation period minus readings at the control site on adjacent normal skin at the start. Each individual served as his/her internal control. Descriptive statistics (t-test) were used.

## Results

### Development of a skin-adapted absorbent core for adult-type briefs

Standard high absorption incontinence products absorb fluid by locking water inside the superabsorbing polyacrylate polymers (SAP). The fluid absorption capacity depends on the polymer neutralization degree and is maximum above pH values of 6.5. In standard polyacrylate superabsorber briefs with a conventional design the surface pH is read at values of 7.08 ± 0.03 (Table 1) when wetted with a salt-containing urine replacement solution. Interposing a fluid acquisition layer containing curled fiber between the top fleece facing the patient's skin and the SAP-containing absorption core (Figure 1A) the surface pH is buffered to 4.58 ± 0.17. Removing the curled fiber layer the pH values inside the diapers did not differ much, 6.58 ± 0.05 for the new design and 6.69 ± 0.32 for the conventional design (Table 1). This creates a pH gradient within the absorbent core, which can be readily seen when a colored pH indicator is used (Figure 1C). This gradient was stable for more than 5 hours (Figure 1D, squared boxes). When a typical urinary micturition pattern was mimicked, the skin-adapted design maintained an acidic surface pH even after repeated wetting. 500 ml of a 0.9% saline solution as urine surrogate were rapidly poured (in less than 30 seconds) over the incontinence brief. The pH was rapidly lowered to 4.5 and drifted to values slightly below 4.0. After 2 hours another 200 ml of the saline solution were applied to the same incontinence brief and again, the pH was rapidly lowered to 4.5 drifting to 4.0 within the next 20 minutes. 5 hours after the first addition of fluid, a final 200 ml of the saline solution were applied and the pH shifted to 4.5 within the first minute drifting very little over the next 20 minutes (Figure 1D, squared boxes). By then, the average absorption capacity was exhausted by approximately 90%. When using standard design incontinence briefs the pH remained in the range of 6.8 -7.0 throughout the whole experiment (Figure 1D, closed circles). The SAP absorbed fluid so efficiently in both diapers, that measurements with a contact pH electrode became unreliable after 20 minutes.

**Table 1:** pH measurement on the surface and inside the absorbent diaper core (3 diapers with triplicate readings, two independent experiments)

	Mean	Standard deviation
Surface pH (incontinence brief - "skin-adapted" design)	4.58	± 0.17
pH inside the absorption core (incontinence brief - "skin-adapted" design)	6.58	± 0.05
Surface pH (incontinence brief - standard design)	7.08	± 0.03
pH inside the absorption core (incontinence brief - standard design)	6.69	± 0.32

### Breathability and stratum corneum moisture after occlusion with different side panel materials

**Figure 2:**
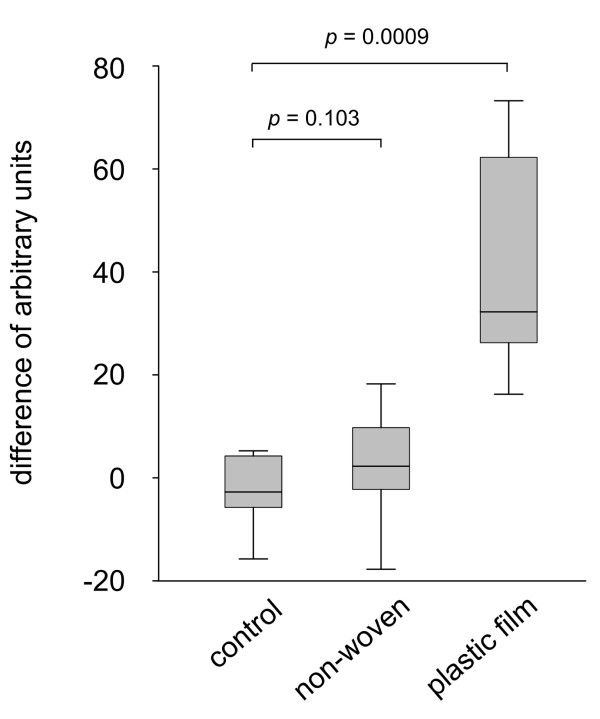
**Stratum corneum hydration after occlusion with different side panel materials**. 4 × 4 cm samples of a non woven fabric and plastic film were fixed on the volar forearm of volunteers for 4 hours. Moisture of the stratum corneum was immediately measured after removal of the materials with a corneometer probe. Corneometer measurements yield relative units which were correlated to intra-individual control values (measurement of adjacent skin without occlusion or treatment). Values are shown as a difference between corneometry readings of the treated skin and untreated skin respectively. An increase in these values indicates an increase in stratum corneum moisture while negative values would indicate water loss and drying-out of the stratum corneum. After the 4 hour incubation period there is a strong increase in the case of plastic film occlusion with an increase in the stratum corneum moisture.

The air-permeability of two standard materials, a non-woven and a plastic film, was measured and the results are shown in Table 2. Plastic film was impermeable, while the non-woven readily allowed pass-through of large quantities of air. To evaluate the effect of breathability 4 × 4 cm samples of each material were fixed on the volar forearm of volunteers for 4 hours. Immediately after the removal of the samples, the moisture of the stratum corneum was measured with a corneometer. Adjacent skin without any applied samples or occlusion served as control. Corneometry measurement values in relative units were calculated as a percentage of the intra-individual corresponding control values. Figure 2 illustrates that after 4 hours the non-woven resulted in a slight increase of the stratum corneum moisture whereas the plastic film caused a substantial increase of 40 arbitrary units.

**Table 2:** Air and water vapor transmission rates of conventional materials for diaper side panels (each determined in tree representative samples, two repeats)

	Breathable, non-woven	Plastic film
Air-permeability [29]	1200 l/m^2^/s	0 l/m^2^/s
Water vapor transmission [30]	3500 g/m^2^/d	0 g/m^2^/d

### Skin symptoms in IAD patients after switch to the skin-adapted brief design

**Figure 3:**
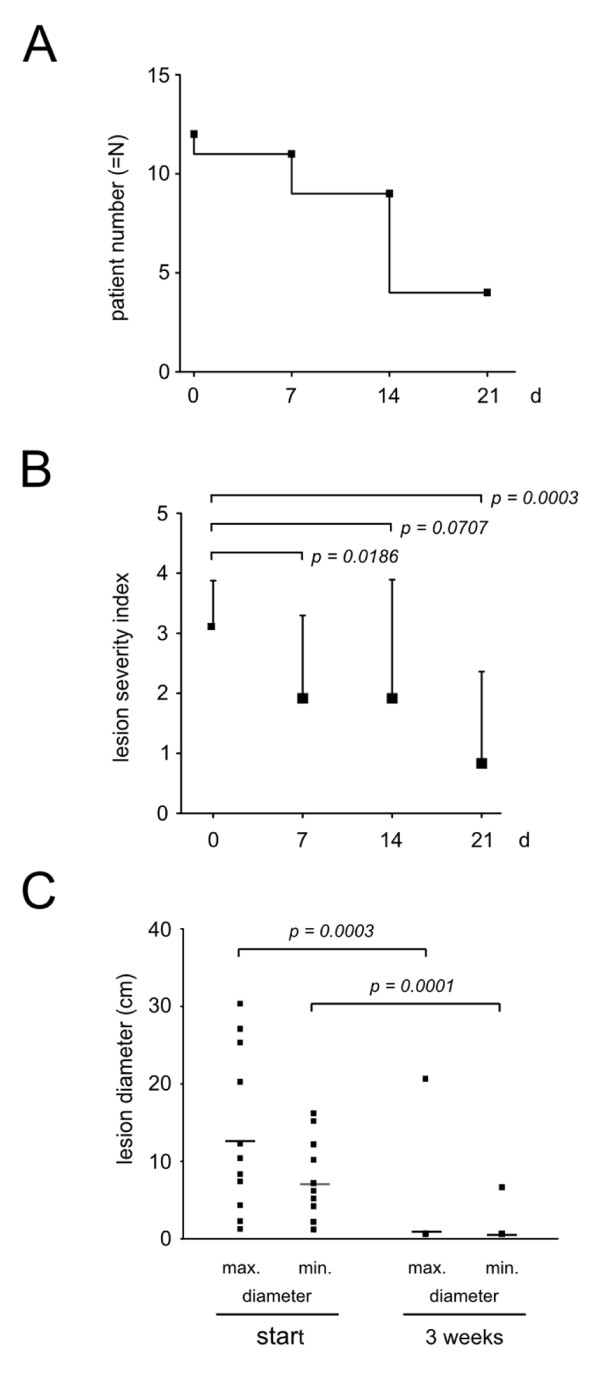
**Skin symptoms in IAD patients after the switch to the skin-adapted brief design**. Skin symptoms in 12 patients with pre-existing IAD were followed for 21 days after incontinence products had been switched to the new skin-adapted version. **A: **Illustration of the number of patients with IAD-associated skin lesions over the 21 day observation period. At the beginning 12 patients had skin lesions. After 21 days, 8 patients were free of skin symptoms while 4 patients were still affected. **B: **The skin severity score (see material and methods) was recorded for the observational period. There was a quick response in the skin severity score after the change to the skin-adapted diaper design within the first week. The score remained rather stable during the second week and a major improvement occurred between days 14 and 21 reflecting the clearing of skin symptoms in 8 patients. In one patient lesions progressed from exuding erythema to lesions in which infection was suspected. **C: **The size of the lesions was compared between the start and at the end of the observational period. 3 patients with disseminated skin lesions had to be excluded from the analysis. In the remaining 9 patients the size of the lesions regressed significantly.

In the course of an institutional switch to the novel, skin-adapted incontinence briefs, skin symptoms were recorded for a 21 day period in 12 patients suffering from IAD. The demographic details and the activity state of the patients are presented in Table 3. In 4 patients, this was the first episode of IAD, in 8 patients it was a recurrent episode (Table 4). The skin lesions consisted of non-exuding infiltrated erythema (9 lesions), exuding infiltrated erythema (8 lesions), pustular rash (5 lesions). Only 3 patients had a single lesion type, in most patients there was coexistence of several types of lesions. The skin care protocol was continued and not altered. After the switch to the skin-adapted diapers, 1 patient became disease-free in the first week (Figure 3A). The skin state improved during the first week as seen by a decrease of the severity score for over 1 point (Figure 3B). During the second week the severity index remained almost constant while 3 patients were healed. At the end of the observation period 8 patients had no skin lesions and the skin score further improved in the remaining. It should be noted though, that in one patient dissemination of the skin lesion was observed. In this patient lesions progressed from exuding erythema to lesions in which infection was suspected and which extended to the whole dorsum, dorsal parts of the upper arms and face. In the remaining 3 patients, the skin lesions had only slightly improved. Also, lesion size improved significantly (Figure 3C). Of note, 3 patients with largely disseminated skin lesions had to be excluded from the analysis. In the remaining 9 patients, the size of the lesions did regress significantly throughout the 3 weeks observational time.

**Table 3:** Demographic details of patients with IAD when switched to the skin-adapted diaper design

	Patient demographics (N = 12)
Age (years)	83.9 ± 6.7
Sex - female (N =)	12
Long-term care (N =)	5
Rehabilitation care (N =)	7

Mobility	

• Mobile (N =)	1
• Reduced mobility (N =)	8
• Bed ridden (N =)	3

**Table 4:** Skin lesion types of patients with IAD at the time of the switch to the skin-adapted diaper design and after the 21 day observation period

Skin lesion type (coexistence of different lesion types possible)		
	**day 0**	**day 21**
	
Infiltrated erythema (non-exuding) (N =)	10	2
Infiltrated erythema (exuding) (N =)	8	1
Pustular rash, follicular erosion (N =)	5	-
Suspected infection (N =)	-	1

First episode (N =)	4	
Recurrent (N =)	8	

## Discussion

Incontinence associated dermatitis (IAD) has long been studied in infants and is termed diaper dermatitis [32,33]. Lesional morphology may vary but common is a challenge by physical, chemical, enzymatic, and microbial factors in the diaper environment. Moist occlusion leads to an increased susceptibility for skin friction and the increased skin hydration as well as an increase in skin pH strongly impair the barrier function of the stratum corneum [32,33]. Eventually, fecal enzymes attack the skin, further adding to the damage. Only in recent years, IAD was addressed and investigated in adult patients [12-14]. From epidermal barrier disruption studies young skin appears less susceptible to external challenges [34]. Moreover, with age the epidermal epidermal barrier recovery takes more time [34], the skin becomes prone to xerosis [35] and the surface pH shifts away from the protective acidic milieu [17]. In addition, overt malnutrition or micronutrient deficiencies, common in the aged population, may further restrict reparative responses to harmful stimuli.

The surface pH plays an important role in epidermal barrier function. While considered to be essential for a physiologic bacterial skin flora, new research highlights the role of an acidic surface pH for the biochemistry of epidermal barrier function and desquamation [17,26]. A number of factors contributes to the altered skin pH in elderly persons. A reduced lipid synthesis rate [36], and reduced moisture binding substances result in xerosis with associated itching [35], and in the case of incontinence, the prolonged exposure to excessive moisture increases the skin pH [6]. Aged skin is more vulnerable and susceptible to irritation [37] and large epidemiological studies from the Netherlands [38] illustrate this in detail. These surveys evaluate the incidence of intertrigo, an inflammatory skin disease, of which the primary pathology is believed to be occlusive skin conditions in skin folds [38]. There is an age-dependent increase in the prevalence of intertrigo from 13% in the over 70 year old population to 17% in the 90 - 94 year old population [38].

To reduce the negative impact of incontinence on epidermal barrier function we describe an optimized diaper design which minimizes occlusion of the skin. The design is creating an acidic surface environment in close contact to the skin while at the same time maintaining optimal pH condition to exhaust the fluid absorption capacity of the gelling superabsorbent polymer inside the incontinence product. This has been achieved by *i*. selection of appropriate raw materials and *ii*. by a three-dimensional design optimization. Even after repeated fluid loading, the surface pH of the optimized incontinence product remained in the acidic, skin-adapted physiological pH range. 20 minutes after each fluid application the surface had become so dry that further pH measurements were impossible. Thus even when wetted repeatedly, a skin-adapted surface pH was maintained while the efficient fluid binding capacity of the superabsorber polymers reduced the duration of surface wetness to a minimum. While our data do not prove a direct effect on the skin pH of patients or volunteers the effect of external hyper-acidfication on the epidermal barrier function has been shown recently [17,26]. We think that an acidic surface of wet incontinence briefs may help to improve to strengthen the epidermal barrier. These effects need further study and are focus of current research. Nevertheless our results can be regarded as first steps in this direction.

Occlusion and over-hydration of the stratum corneum is known to weaken the epidermal barrier. Therapeutically this is employed to increase the potency of topical corticosteroid preparations [39]. In the presence of irritants, however, the effect can be deleterious as observed from i.e. patients with hand dermatitis having to wear protective gloves. In the context of diapers it seems obvious to select side panels with a high air-permeability. When compared with film materials commonly utilized we found differences in breathability between different fabrics. Some diapers have plastic films, sometimes micro-perforated, onto which a non-woven is bonded. The haptic aspect is like a non-woven, nevertheless air-permeability is very low (micro-perforated films up to 50 l/m2/s, unpublished data) or non-existing (plastic film backing, Kesselmeier et al., unpublished data). Nevertheless, many patients prefer film-type diapers as they value a reduced air circulation which may retain unpleasant odors within the briefs.

In our setting, the occurrence of IAD initially was clustered in one institution while at the same time other institutions of the Assistance Paris reported no increased frequencies. Analysis of possible triggers and care protocols did not identify a particular, precipitating factor. Moreover, in all institutions defined skin care protocols had been established and were followed to minimize the negative impact of incontinence on the barrier function of the skin. The skin care protocols consisted of daily cleansing of the perineal zone with water or with water and detergents after defecation, drying of the skin by carefully avoiding skin friction and application of a protective skin cream. Despite these precautions IAD still occurred. When patients were switched to the skin-adapted incontinence product design, the skin lesion severity score responded with a fast decrease in the first week. However, healing of the skin lesions occurred in 8 patients only between days 14 and 21 possibly reflecting the reduced repair competence in aged patients and the continued persistence of incontinence as a precipitating factor.

Our data have limitations. As mentioned above, the effect of an acidic surface pH on the diapers can significantly support and normalize the physiology of the aged epidermal barrier. To prove this additional research is needed and currently focus of our group. We are also aware of the limitations of our test methods. Urine surrogate solutions do not reflect the complexity of natural urine. Depending on the dietary intake acid and basic equivalents are excreted by the kidneys into the urine and cause variations in urinary pH and composition. Still, these solutions offer the advantage of standardization and are routinely employed to resolve complexity. Corneometry is only an indirect measurement of epidermal barrier hydration [27]. However, the wide utilization and the extensive validation in cosmetic science permits the adaptation of this non-invasive technique and allows interpretation within the limits of the technique. Finally, we would like to discuss our clinical data critically. From a practical point the creation of a clinical structure to bring IAD lesions to the attention of dedicated specialists who could consult dermatologists in case of need was most important for the successful treatment (Beguin et al., manuscript in preparation). This structure, together with intensive routine documentation of skin lesions, was a prerequisite to condense the clinical data into a scoring system like ours which can illustrate the overall severity and healing progress. We need to caution that this score is not validated at this point and the clinical data are only descriptive. Much larger clinical studies are needed, yet, the encouraging results reported here provide a basis for future work on the preventive potential of the new brief design on the occurrence of IAD.

## Conclusions

We conclude, that it is possible to design absorbent incontinence products which can help to support the epidermal barrier function. This can be achieved by creating an acidic surface pH on the absorbing part of the diaper. In addition, the use of highly breathable materials in side panels reduces the occlusive effect of diapers and can minimize stratum corneum over-hydration. In combination, these properties can reduce the negative impact of incontinence on skin health.

## Abbreviations

IAD: incontinence associated dermatitis; SAP: superabsorbent polymer

## Competing interests

AMB, EM-P, CG, A-MH-L and SS are hospital employees and declare no conflict of interest. VH, PZ, MS, RK, FH and HS are employees of Paul Hartmann AG.

## Authors' contributions

AMB, EM-P, CG, A-MH-L and SS contributed the clinical data and helped writing the manuscript VH conducted the experiments shown in Figure 1 and contributed to the writing of the manuscript. PZ and MS measured skin hydration after application of the different materials as shown in Figure 2. RK and FH were involved in optimizing the absorbent core design and contributed to the writing of the manuscript. HS designed the study and wrote the manuscript. All authors read and approved the final manuscript

## Pre-publication history

The pre-publication history for this paper can be accessed here:

http://www.biomedcentral.com/1471-2318/10/86/prepub
